# Calcium signaling: breast cancer’s approach to manipulation of cellular circuitry

**DOI:** 10.1007/s12551-020-00771-9

**Published:** 2020-12-18

**Authors:** Stephen JP Pratt, Erick Hernández-Ochoa, Stuart S Martin

**Affiliations:** 1 Program in Biochemistry and Molecular Biology, University of Maryland School of Medicine, Baltimore, MD USA; 2 Department of Physiology, University of Maryland School of Medicine, Baltimore, MD USA; 3 Marlene and Stewart Greenebaum NCI Comprehensive Cancer Center, University of Maryland School of Medicine, 655 W. Baltimore Street, Bressler Research Building, Rm 10-020 D, Baltimore, MD 21201 USA; 4 Department of Biochemistry and Molecular Biology, University of Maryland School of Medicine, Baltimore, MD USA

**Keywords:** Breast cancer, Calcium signaling

## Abstract

Calcium is a versatile element that participates in cell signaling for a wide range of cell processes such as death, cell cycle, division, migration, invasion, metabolism, differentiation, autophagy, transcription, and others. Specificity of calcium in each of these processes is achieved through modulation of intracellular calcium concentrations by changing the characteristics (amplitude/frequency modulation) or location (spatial modulation) of the signal. Breast cancer utilizes calcium signaling as an advantage for survival and progression. This review integrates evidence showing that increases in expression of calcium channels, GPCRs, pumps, effectors, and enzymes, as well as resulting intracellular calcium signals, lead to high calcium and/or an elevated calcium- mobilizing capacity necessary for malignant functions such as migratory, invasive, proliferative, tumorigenic, or metastatic capacities.

## Ja Kalzium, das ist alles

When calcium ions (Ca^2+^) flow into a cell, they can bind to calcium-binding proteins to ultimately initiate cell functions. Paradoxically, however, this versatile single element universally participates in almost every single cell process: death (Boehning et al. 2003; Orrenius et al. 2003), cell cycle (Colomer et al. 1994; Kahl and Means 2004), division (Rasmussen and Means 1989), migration (Brundage et al. 1991; Giannone et al. 2004; Hahn et al. 1992; Yang and Huang 2005), invasion (Kato et al. 2007; Sun et al. 2014), metabolism (Cardenas et al. 2010), differentiation (Carey and Matsumoto 1999; Hennings et al. 1980; Holliday et al. 1991), autophagy (Cardenas et al. 2010; Medina et al. 2015), and transcription (Dolmetsch et al. 1998, 2001), as well as participating in many specialized cell functions: angiogenesis (Dragoni et al. 2011), fertilization (Miao et al. 2012; Saunders et al. 2002; Steinhardt et al. 1977), insulin secretion (Grodsky and Bennett 1966; Prentki and Wollheim 1984), synaptic transmission (Brose et al. 1992; Fernandez-Chacon et al. 2001), muscle contraction (Gergely et al. 1993; Sorenson et al. 1995), and immune response (Bhakta et al. 2005). This is perhaps why scientists quote the Nobel prize winning Otto Loewi’s proclamation, “Ja Kalzium, das ist alles” (Carafoli 2002), which literally translates to “Yes calcium, that’s all,” but instead is often interpreted as “calcium is everything” or “calcium is universal” (Brini and Carafoli 2000; Kaestner 2013).

The paradox of calcium in biology is that the divalent cation remains unchanged yet can still achieve specificity in initiating various cell processes. In other words, when calcium enters a cell, it is not bringing forth cell death, cell cycle, division, migration, invasion, metabolism, differentiation, autophagy, and transcription all at once, rather each process can be individually initiated by calcium while the other calcium-sensitive functions remain at rest. Before we discuss how calcium can discriminate between these various cell functions, let us begin with a simple binary (on/off) introduction to calcium signaling.

## Introduction to calcium signaling

The plasma membrane is a lipid bilayer barrier that separates the outside and inside of the cell. However, contained within the cell are separate lipid barriers that serve to further compartmentalize cell contents. These intracellular membrane-bound areas, known as organelles, perform different cell functions and include the nucleus, Golgi apparatus, endoplasmic reticulum (ER), mitochondria, and various vesicles. The general non-membrane bound areas within the cell are collectively known as the cytoplasm. Calcium ions are differentially concentrated across these cell membranes between the outside of the cell, the cytoplasm, and within organelles. This separation of calcium is key to calcium signaling.

Calcium is a metallic element and fifth in abundance in the earth’s crust (2015). In the human body, most of the calcium exists in both a bound-form (bones, teeth or bound to extra- and intracellular proteins) and an ionized free form. When considering cells at rest, free calcium is present in relatively high concentrations outside the cell (~ 1.3 mM) (Kratz et al. 2004), and with respect to mammary glands, there is ~ 10 mM total calcium (Neville 2005) and ~ 2–4 mM free calcium in human milk (Neville et al. 1994). In contrast, free calcium is at very low concentrations in cytoplasm (~ 0.05–0.15 mM (McDonough and Button 1989; Ratto et al. 1988)). While resting free calcium concentrations within the cellular organelles vary (nucleus (~ 0.03–0.2 mM, (al-Mohanna et al. 1994; Brini et al. 1993; Ikeda et al. 2003; Przywara et al. 1991; Williams et al. 1985)), Golgi apparatus (~ 0.3 mM (Pinton et al. 1998)), ER (~ 0.5–0.7 mM (Launikonis et al. 2005)), mitochondria ~ 0.2 mM (Ivannikov and Macleod 2013)), lysosomes (~ 0.4 mM (Christensen et al. 2002)) (note that these are approximations)), there is still a concentration gradient between the cytoplasm and Golgi/ER/lysosomes, similar to that between the cytoplasm and extracellular space. The high vs. low concentrations are actively maintained by the cell using different transport mechanisms such as adenosine triphosphate (ATP)-driven calcium pumps that drive calcium within the ER (via sarco-/endoplasmic reticulum Ca^2+^-ATPase, SERCA) or Golgi apparatus (via Secretory Pathway Ca^2+^-ATPase, SPCA), or drive calcium outside of the cell (via plasma membrane Ca^2+^-ATPase, PMCA and sodium–calcium exchanger, NCX). In exchange for ATP consumption, the stored energy that is present in the ion’s electrochemical potential gradient can be utilized. If permitted across cell membranes, ions will passively move down an electrochemical potential gradient (from high to low) until equilibrium is achieved. Thus, calcium will flow from the outside of the cell or from within internally membrane-bound compartments (the ER is the major store) to the cytoplasm or even into the nucleus and mitochondria. Even though ATP pumps continually drive calcium against its electrochemical potential gradient (to areas of high calcium concentrations), equilibrium cannot practically be achieved; however, a steady state of cytoplasmic and organelle calcium concentrations can be.

**Fig. 1:**
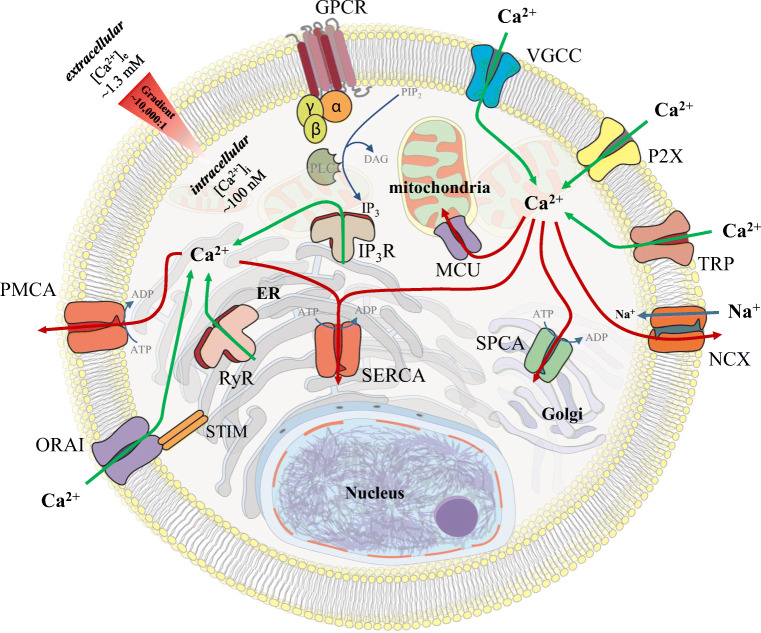
Mechanisms for cellular calcium mobilization. The plasma membrane and intracellular membrane proteins responsible for calcium mobilization are shown. Calcium ions are differentially concentrated across cell membranes between the outside of the cell, the cytoplasm, and within organelles. This separation of calcium is key to calcium signaling. In a resting cell, high free calcium concentrations are maintained outside the cell (~ 1.3 mM), while free calcium is at very low concentrations in cytoplasm (~ 0.1 mM), establishing an ~ 10:000:1 gradient across the plasma membrane. Resting free calcium concentrations within the cellular organelles vary (nucleus (~ 0.3–0.2 mM), Golgi apparatus (~ 0.3 mM), ER (~ 0.5–0.7 mM), mitochondria ~ 0.2 mM), lysosomes (~ 0.4 mM) (note that these are approximations)). Calcium can enter the cell into the cytoplasm through plasma membrane ion channels (VGCCs, P2X, TRPs, ORAI) or ER membrane channels (IP3Rs and RyRs). Alternatively, cytosolic calcium can be depleted through mitochondrial calcium uptake (via MCU channels), ATP-driven pumps (PMCA, SERCA, SPCA), or the sodium–calcium exchanger (NCX)

Calcium signaling refers to the mobilization of calcium ions down these electrochemical potential gradients and this can be initiated in various ways (for in depth reviews on calcium signaling, see Berridge et al. 2000, 2003; Carafoli 2002; Carafoli et al. 2001; Clapham 2007, and see mitochondrial (Rizzuto et al. 2012) and nuclear (Bootman et al. 2009) calcium signaling focused reviews). There are two major classes of membrane bound proteins that permit calcium mobilization, ion channels and G-protein coupled receptors (GPCRs), which can be present on the cell’s plasma membrane and on organelle membranes (Fig. 1). Ion channels, once activated, open their central pore regions allowing calcium ions to mobilize through the channel. In contrast, activated GPCRs typically release ER calcium using lipid signal transduction to target ER ion channels. The resulting movement of calcium ions to the cytoplasm increases the calcium concentration and the positively charged ions are free to bind to oppositely charged counterparts (see the review on calcium-binding domains and motifs (Carafoli et al. 2001)), known as buffers and effectors (further reviews on buffers (Schwaller 2010) and effectors (Berridge et al. 2003) are available). Most of this cytosolic calcium is bound by buffers, which can limit or tune the cytoplasmic calcium signal and thus limit/tune the calcium bound by effectors, depending on the amount, localization, and type of buffer (i.e., fixed vs. mobile) present within the cell. In contrast, calcium-bound effectors play a more direct role in cell function by initiating downstream signaling (i.e., cell death, cell cycle, division, migration, invasion, metabolism, differentiation, autophagy, or transcription). Calcium signaling is eventually finalized as calcium ion channels deactivate and close, thus no longer permitting calcium passage into the cytoplasm. The calcium pumps simultaneously restore low cytoplasmic calcium concentrations and high ER/Golgi calcium concentrations by pumping calcium outside of the cell and into the organelle stores respectively. An additional mechanism by which calcium is removed from the cytoplasm is through the sodium–calcium exchanger (NCX) and is particularly notable in excitable cell types. Buffers and effectors then become unbound by calcium as the concentration of cytoplasmic calcium continues to fall to resting concentrations, and thus effectors inactivate and effector-mediated downstream signaling ceases.

This binary initiation/termination of calcium signaling explanation is the basis of understanding calcium signaling. However, the complex versatility of calcium ions in the wide variety of cell functions, as outlined above, is not possible in a binary world. Otherwise, increases in cytosolic calcium would simultaneously trigger all cell functions. Rather, the versatility of calcium ions is possible on a spectrum of calcium signals, which is the topic of the next section. Calcium signals can be big or small, be fast or long lasting, and be global or local. These amplitude, frequency, and spatial spectra ultimately regulate the discrimination between different cell functions and give the cell the tools to appropriately “choose” its fate.

## Amplitude, frequency, and spatial modulation

One question when considering calcium ion flux into the cytoplasm is, how many ions enter? The amplitude of the calcium signal measured yields the answer (through the integral of the curve). The higher the amplitude, the more calcium occupies the cytoplasm, the lower the amplitude, less calcium. In a process known as amplitude modulation (AM) (Berridge 1997), these different amplitudes, or amounts of global cytosolic calcium, trigger different downstream signaling responses. For example, in B lymphocytes, changes in ~ 0.2–0.4 mM of calcium results in activation of the transcription factor, nuclear factor of activated T cells (NFAT) (i.e., nuclear translocation) without activating the nuclear factor kappa-light-chain-enhancer of activated B cells (NFκB) or c-Jun N-terminal kinase 1 (JNK1) (i.e., IκBα degradation and JNK1 phosphorylation), whereas ~ 0.4–0.6 mM of calcium is required to activate NFκB/JNK1 (Dolmetsch et al. 1997). In this way, different downstream signaling pathways can be activated based on the amount of calcium permitted into the cell. This can be possible through an affinity-based mechanism whereby higher affinity calcium-binding proteins are activated with lower calcium concentrations and lower affinity calcium-binding proteins are activated with higher calcium concentrations, and/or possible via fractional occupancy-based mechanisms whereby proteins with one calcium-binding site are activated with lower calcium concentrations and proteins with up to four calcium-binding sites are activated with higher calcium concentrations (Parekh 2011). However, the limitation is that an increasing calcium signal will not activate proteins of differing affinities in a completely independent manner but will rather simply activate them sequentially. In other words, 0.6 mM of calcium will activate both NFAT and NFκB/JNK.

Calcium can also differentially activate downstream signaling pathways based on the temporal characteristics of the calcium signal. In a process known as frequency modulation (FM) (Berridge 1997), transient and repetitive elevations in cytosolic calcium, known as oscillating calcium signals, can determine different protein activation (Samanta and Parekh 2017). For example, the calcium-binding protein kinase C (PKC) can be partially activated with low-frequency calcium spikes, whereas maximal activation of PKC occurs during high-frequency calcium spikes (Oancea and Meyer 1998). Similarly, calcium- and calmodulin-dependent protein kinase II (CAMKII) (De Koninck and Schulman 1998) or calcium-sensitive mitochondrial dehydrogenases (CSMDHs) (Hajnoczky et al. 1995) can show greater activity with increasing frequency of calcium exposure. Oscillations can even discriminate between proteins within the same cell, low-frequency oscillations activate NFκB, while high frequencies activate NFAT (Dolmetsch et al. 1998). At least for NFAT, these oscillation-dependent mechanisms intriguingly seem to be entirely independent of total average calcium in the cell (i.e., amplitude) and rather specifically sensitive to a certain frequency of oscillations (Dolmetsch et al. 1998; Li et al. 1998).

Calcium-dependent activation of proteins can be further distinguished via spatial restriction of calcium signals (for more in depth reviews, see Parekh 2011; Samanta and Parekh 2017), which is in contrast to the global cytosolic calcium elevations discussed up until now, where the total cytosolic space is theoretically occupied. When considering ion flux through a single channel into the cytoplasm from the plasma membrane, free calcium concentrations indeed decrease with increasing distance from the channel (Neher 1998; Rizzuto and Pozzan 2006), which is largely due to the presence of buffers that limit the range of calcium diffusion. For example, during fast calcium influx, differences in calcium concentrations between the closest region to the inner leaflet of the plasma membrane, called the subplasma membrane, and the cytoplasm range from ~ 0.4 to 0.8 mM (Nagai et al. 2004; Pinton et al. 2002). Even at rest, there seems to be some graded calcium concentrations between the subplasma membrane and the cytoplasm (0.8 vs. 0.3 mM, respectively) (Nagai et al. 2004). Importantly, these microdomains of larger calcium concentrations relative to the cytoplasm have functional consequences. For example, cAMP response element-binding protein (CREB) phosphorylation via CAMK relies on calcium concentrations spanning 1–2 μm from the subplasma membrane (Deisseroth et al. 1996). Similarly, local signals can specifically trigger activation of transcription factors c-Fos (Di Capite et al. 2009), signal transducer and activator of transcription (STAT) 5 (Ng et al. 2009), and NFAT (Kar et al. 2011, 2016). These domain-dependent signaling pathways rely on the localization of effectors near calcium channels; therefore, re-localization of effectors away from the subplasma membrane would inhibit these pathways. Moreover, removal of cell buffering capacity may activate effectors not tethered near subplasma regions. Finally, spatial restriction of calcium signals independent of buffers are possible through elementary calcium events localized near calcium channels. The elementary calcium events are either derived from single ion channels, known as blips from inositol 1,4,5-trisphosphate receptors (IP_3_Rs) and quarks from ryanodine receptors (RYRs), or when groups of 10–20 channels collectively release calcium, called puffs (IP_3_R), and sparks (RYR) (Berridge 1997). Puffs and sparks release up to ~ 0.6 mM of calcium within a spatial spread of up to ~ 7 μm which can ultimately lead to larger calcium waves in the cell (Niggli 1999; Thomas et al. 2000).

These three means of calcium modulation are “clever” ways of getting around the calcium signaling paradox. The versatile calcium ion can be used for activating the diversity of cell functions without changing the ion itself, but rather changing the characteristics (amplitude/frequency modulation) or location (spatial modulation) of the signal. It is important to note, now that the characteristics of calcium signaling have been introduced, that these three modulation mechanisms are not necessarily mutually exclusive. For example, consider a low-affinity effector which requires a high concentration of ions for activation, but only a concentration of ions below the threshold for activation have entered a cell. Simply changing the effector’s location to near a channel where local calcium concentrations relative to the global cytosolic calcium is high enough would ensure activation, or vice versa. Thus, two or more means of modulation can be utilized in sync to add additional regulation of downstream signaling. Further considering all three, and that each sits on a spectrum of magnitude, gives the cell exponential combinations of tuning intracellular calcium signaling. These are the possibilities needed for an investigator to recognize when experimenting with calcium signaling and determining how calcium will affect the cell.

## Calcium channels, buffers, and effectors

Now that the versatility of the calcium ion has been explained through spatial, amplitude, and frequency modulation, how calcium enters the cell and what it can do once inside the cell will now be discussed. There are many calcium channels, GPCRs, buffers, effectors, pumps, and calcium-sensitive enzymes that contribute the initiation of calcium signaling, the cell response, and the decay of the calcium signal. Berridge, Bootman, and Roderick have termed these elements as a cell’s “calcium signaling toolkit” (Berridge et al. 2003). As mentioned above, there are two major protein families that control the initiation of calcium signaling inside the cell: G-protein coupled receptors (GPCRs) and ion channels. GPCRs and ion channels are briefly introduced in this section, but there are extensive reviews available for the reader (Berridge et al. 2003; Tsien and Tsien 1990).

Ion channels are membrane bound proteins that form a central pore that can pass (i.e., conduct) ions when activated and open, or block ion flux when inactivated or deactivated and closed (inactivation describes a channel that has stopped conducting ions but has not fully closed, while deactivation describes a channel that has full closed). Ion conduction through an open channel is possible when there is an electrochemical potential gradient created by differential ion concentration across cell membranes, as discussed above. Some ion channels specifically permeate only one type of ion, such as potassium, sodium, calcium, or chloride; however, others can be more non-selective and can permit multiple ions. For example, transient receptor potential (TRP) channels can be permeable to multiple ions (reviewed in (Bouron et al. 2015)). The specificity in permeability not only is achieved through the charge of the residues lining the central pore region and its compatibility with an oppositely charged ion but is also governed by the geometry of the pore and its compatibility with ions of a specific size (Sun et al. 1997). These characteristics of protein structure, what is known as the selectivity filter of ion channels, are achieved on a molecular level via backbone carbonyls and specific amino acids. These specific structures of ion channels have evolved not only for specificity in ion selection (Doyle et al. 1998) but also for optimal rapid conductance of ions when the channels are open (Morais-Cabral et al. 2001). Ions are stabilized and dissolved in solution through hydration by water molecules; however, only ions and water molecules pass through channels in single file, and therefore, ions must shed their water molecules as they move through the pore (Doyle et al. 1998). Ions are then stabilized by the charged central pore as the ion passes through and water on the opposite side of the channel then re-hydrates the ion. While this may seem like a time-consuming process, ion permeation through channels is rather quite rapid because it minimizes energy constraints (Morais-Cabral et al. 2001) when compared with other forms of ion flow such as transport through pumps.

Ion channel activation can occur through a large variety of mechanisms (Fig. 1). Voltage-gated calcium channels (VGCCs) open their central pore regions (i.e., are gated) in response to changes in membrane voltage and specifically permeate calcium ions. VGCCs are typically present in excitable cells such as neurons and muscle and play roles in synaptic transmission and muscle contraction. Purinergic receptor ion channels, known as P2X channels, bind nucleotides (AMP, ADP, ATP) and permeate calcium (Valera et al. 1994). Inositol 1,4,5-trisphosphate receptors (IP_3_Rs) are present on the endoplasmic reticulum (ER) and permeate calcium upon the binding of inositol 1,4,5-trisphosphate (IP_3_). Ryanodine receptors (RYR) are present in muscle on the sarcoplasmic reticulum (SR) and in non-excitable cells on the endoplasmic reticulum (ER) and are activated by calcium in a process known as calcium-induced calcium release. RYRs can also be activated by depolarization-induced calcium release in skeletal muscle. In a process known as store-operated calcium entry (SOCE) (but has in the past been referred to as capacitative calcium entry), stromal interaction molecule (STIM)/ORAI complexes serve to replenish intracellular calcium stores after calcium release, from locations such as the ER, since some of the calcium gets pumped to the extracellular space instead of entirely back into internal stores. While both STIM/ORAI form what is known as the calcium release-activated calcium (CRAC) channel, ORAI is the pore-forming subunit that resides in the plasma membrane, while STIM is an ER calcium-sensing and ORAI activating protein in the ER membrane. Finally, there are the transient receptor potential (TRP) family of ion channels that range in selectivity but generally permit calcium and magnesium ions to pass through their central pore regions. TRP channels are composed of seven subfamilies: TRPC (canonical), TRPV (vanilloid), TRPM (melastatin), TRPP (polycystin), TRPML (mucolipin), TRPA (ankyrin), and TRPN (NOMPC-like) (reviewed in (Zheng 2013)). A very interesting family of channels, TRP channels can be activated in response to cold/menthol, stretch-activation, pH, calcium, and voltage (reviewed in (Clapham et al. 2001; Zheng 2013)).

In contrast to ion channels, GPCRs do not facilitate calcium movement through a central pore, but rather indirectly activate calcium signaling. GPCRs are membrane-bound proteins that are typically activated by ligands such as hormones, peptides, and neurotransmitters, but there are other activation mechanisms such as pH or mechanical stimuli. GPCRs are coupled to the G-proteins Gα, Gβ, and Gγ on the cytoplasmic side (Gα and Gγ are tethered to the plasma membrane by lipid anchors). This complex represents the inactivated state of GPCRs. Once activated, the exchange of GTP with GDP on the Gα subunit occurs and the GTP-bound Gα and Gβ/Gγ dimer decouple from the receptor and each other. The GTP-bound Gα and Gβ/Gγ dimer move on separately to activate downstream signaling. For calcium, it is the Gα subunit that activates phospholipase C (PLC) which in turn converts the membrane lipid phosphatidylinositol 4,5-bisphosphate (PIP_2_) to diacylglycerol (DAG) and inositol 1,4,5-trisphosphate (IP_3_). IP_3_ in turn diffuses from the plasma membrane and binds to and opens IP_3_R channels located on the ER resulting in calcium efflux from the ER to the cytoplasm. Then Gα unbinds GTP and re-binds GDP, and Gα and Gβ/Gγ reassociate with each other as well as with the GPCR.

Once calcium has entered the cell, it quickly associates with calcium-binding proteins, which contain negatively charged and geometrically compatible protein domains or motifs (i.e., structures) that are capable of binding calcium (reviewed in (Carafoli et al. 2001)). For example, the EF-hand motifs bind two calcium ions per molecule. EF-hand motif containing calcium-binding proteins include calmodulin and the S100 protein family. There are also non-EF-hand calcium-binding proteins such as annexins, gelsolin, calreticulin, and those with C2-domains (protein kinase C is a very common enzyme with a C2-domain). Calcium-binding proteins can be generally categorized into two broad categories: buffers and effectors/sensors. While buffers/effectors are briefly introduced in the next paragraph, there are extensive reviews on buffers (Schwaller 2010; Yanez et al. 2012) and effectors (Berridge et al. 2003; Carafoli et al. 2001; Yanez et al. 2012) available for the reader.

Buffers quickly chelate calcium ions that enter the cell and ultimately can change the amplitude, frequency, and spatial characteristics of the free cytosolic calcium available to bind to effector proteins. The role of buffers in spatial modulation has already been outlined, and one can imagine how buffers can similarly affect the amplitude and frequency of calcium signaling. Therefore, buffers limit the amount of calcium freely available to a cell to activate functions (i.e., cell death, cell cycle, division, migration, invasion, metabolism, differentiation, autophagy, or transcription). Major buffers include parvalbumins, calbindin-D9k, calbindin-D28k, calretinin, calreticulin, calnexin, calsequestrin, and GRP78/94. In contrast, effectors/sensors serve to initiate downstream cell signaling pathways which ultimately lead to turning on cell functions. Several effectors have already been introduced with respect to activation under amplitude, frequency, and spatial modulation. Major effectors include the calmodulin and S100 protein families, but there are also troponin C, synaptotagmin, the annexin protein family, myosin light chain kinase, protein kinase C family, calcineurin, calmodulin-dependent protein kinases (CAMKs), calpain proteases, nitric oxide synthases, nuclear factor of activated T cell transcription factor family, cyclic AMP response element-binding protein (CREB) transcription factor, and downstream regulatory element modular (DREAM) transcription factor.

## Methods for measuring intracellular calcium

**Fig. 2:**
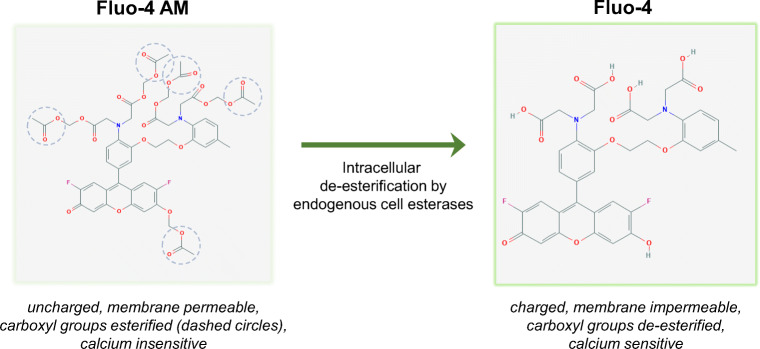
Mechanism of Fluo-4 cellular uptake. The most common method for measuring intracellular calcium is through fluorometric microscopy-based visualization of calcium using calcium-binding fluorescent indicators such as Fluo-4. Dye structures for Fluo-4 AM and Fluo-4 were derived from https://pubchem.ncbi.nlm.nih.gov/. The Fluo-4 AM molecule contains ester groups that renders the molecule uncharged and can thus freely diffuse across the cell plasma membrane into the cell (left). Once inside the cell, intracellular esterases cleave these groups from the molecule, and the molecule becomes charged and is thus impermeable to the cell membrane (right). The trapped intracellular charged Fluo-4 is also sensitive to binding calcium and will become brightly fluorescent only in the calcium-bound state

The most common method for measuring calcium, and especially calcium inside cells, is through microscopy-based visualization of calcium using calcium-binding fluorescent indicators. These indicators can be either exogenous (artificially introduced within the cell) or endogenous (genetically introduced within the cell) (Rudolf et al. 2003; Tsien 1980, 1981). Exogenous indicators are commonly referred to as dyes and include green fluorescing Fluo-4, red fluorescing Rhod-2, and UV-excited Fura-2, and Indo-1. These dyes are introduced into the cell by diluting them into the extracellular media for an incubation time. By way of ester groups, they remain as uncharged molecules that can therefore freely diffuse across the cell plasma membrane. However, once inside the cell, intracellular esterases cleave these groups from the molecule, they become charged, are thus impermeable to the cell membrane, and become trapped inside (Fig. 2). Moreover, the charged molecules become sensitive to binding calcium and will become brightly fluorescent only in the calcium-bound state.

Exogenous dyes fall into two general categories, ratiometric and non-ratiometric dyes. Non-ratiometric dyes fluoresce in one color when bound by calcium. As calcium concentrations increase in the cytoplasm during calcium mobilization, calcium binds to the dye, the calcium-bound dye can then fluoresce, and the fluorescent light can be captured by a light-detecting device (photodiode, photomultiplier tube, or camera such as CCD) attached to a microscope during this process. Therefore, the changes in calcium signaling (i.e., increasing calcium, decreasing calcium, or calcium oscillations) can be visualized because changes in fluorescent light correlate with changes in calcium concentration. Calcium signaling can be qualitatively measured (i.e., relative amplitude, time course) using calculations for Δ*F*/*F* = (*F* − *F*_0_)/*F*_0_, where *F* = the fluorescence of the calcium dye when calcium signaling is occurring, and *F*_0_ = baseline fluorescence of the calcium dye before the initiation of calcium signaling. Non-ratiometric dyes are easy to use and do not require very special equipment. However, there are limitations to consider. When comparing sample to sample in an experiment, a user cannot exactly control the amount of calcium dye that gets into a single cell or on a cell to cell basis. This is a problem, because more dye will result in more fluorescence, which artificially reflects more calcium signaling. Therefore, one might be artificially recording differences in calcium signaling between samples. Yet, calculations for Δ*F*/*F* generally allow one to compare relative changes in calcium signaling between samples, since the Δ*F*/*F* = (*F* − *F*_0_)/*F*_0_ equation normalizes for sample to sample differences in dye concentration. The other major limitation is that users cannot measure exact calcium concentrations within a cell, or calcium concentrations between sample to sample, and they can only measure relative changes in calcium during calcium signaling (because of normalization steps). However, semi-quantitative approaches are available for non-ratiometric dyes (Maravall et al. 2000). Ratiometric dyes on the other hand can allow for one to directly compare calcium concentrations within a cell and between samples. This is because these dyes change color or excitation spectrum as calcium is bound to the dye. Therefore, users can compare the ratio of background fluorescence of the dye in one color/spectrum and changes in calcium concentration in another color/spectrum, within the same sample. In this way, differences in dye concentration can be normalized within a sample. Furthermore, the calcium concentration can then be extrapolated after the dyes are calibrated to known calcium concentrations.

Finally, there are endogenous calcium indicators that come from genetically encoded proteins. These have been engineered and are artificially introduced into cells, but once the DNA encoding the calcium indicator has been introduced, the protein-based indicators are produced by the cell from DNA and can remain intracellular. For example, GCaMP6 is a combination of green-fluorescent protein and calmodulin, which makes it a calcium-sensitive protein-based indicator that can be used to measure intracellular calcium signaling much like that of Fluo-4 (Chen et al. 2013).

## Calcium signaling and cancer

So far, the detailed complexities of calcium signaling have been explained under normal contexts. However, as with many other cell signaling pathways, cancer cells can disfigure what is considered “normal” and change the prevailing choreography of calcium channel/receptor activation, intracellular calcium signaling, and downstream calcium-sensitive signaling. Moreover, overall calcium signaling modifications can be the result of adjusting any step of the calcium signaling pathway; it is possible for cancer cells to change upstream channel/receptor activation signals, cytoplasmic/organellar calcium concentrations available for calcium release, or the state or number of calcium channels, GPCRs, buffers, and effectors. Through these mechanisms, a cancer cell may overactivate, inhibit, or otherwise adjust its intracellular calcium signaling, which might serve as a survival advantage to the cell. The literature and current knowledge on how calcium signaling pathways are altered in breast cancer will be reviewed and the outcome of these changes with respect to cell function and survival will be discussed.

Altered calcium handling has indeed been implicated in cancer and there are many reviews available (Anguita and Villalobo 2018; Azimi and Monteith 2016; Azimi et al. 2014; Berchtold and Villalobo 2014; Bhargava and Saha 2019; Bong and Monteith 2018; Busselberg and Florea 2017; Chalmers and Monteith 2018; Cross et al. 2014; Cui et al. 2017; Deliot and Constantin 2015; Frisch et al. 2019; Grimm et al. 2018; Haworth and Brackenbury 2019; Hempel and Trebak 2017; Humeau et al. 2018; Iamshanova et al. 2017; Kadio et al. 2016; Kim and Wysolmerski 2016; Makena and Rao 2020; Maly and Hofmann 2018; Marchi and Pinton 2016; Martinez-Delgado and Felix 2017; Mignen et al. 2017; Missiroli et al. 2017; Mo and Yang 2018; Monteith et al. 2007; Monteith et al. 2017; Orrenius et al. 2003; Pierro et al. 2019; Prevarskaya et al. 2010, 2011; Ritaine et al. 2017; Roberts-Thomson et al. 2019; Sallan et al. 2018; So et al. 2019; Sterea and El Hiani 2020; Stewart et al. 2015; Tajbakhsh et al. 2018; Terrie et al. 2019; Tsai et al. 2015; Venkateswaran et al. 2018; Villalobos et al. 2017; Xu et al. 2018), including some excellent breast cancer-focused reviews (Azimi et al. 2014; Cross et al. 2014; Lee et al. 2006; So et al. 2019; Tajbakhsh et al. 2018). The data from the literature can be generally categorized into three groups. First, there are quantified cancer-mediated changes in the components of the calcium signaling toolkit: expression differences of calcium channels, pumps, GPCRs, and calcium-sensitive proteins from patient samples and cancer cell lines are measured against normal tissue or non-tumorigenic cell lines. Measured expression changes are often correlated with tumor grade, patient outcome, or tumorigenic capacity of cancer cells. Second, overexpression or knockout of components of the calcium signaling toolkit are used to enhance or inhibit the tumorigenic behavior of cells, which help determine how observed expression changes seen in patients and cancer cell lines affect cancer in a more mechanistic way. Third, there are direct measurements of intracellular calcium signals under different contexts, (i.e., in relation to normal vs. cancer cells or overexpression/knockout cells). It is useful to organize the data in this way as an attempt to more comprehensively understand the detailed mechanisms underlying cancer-mediated changes in calcium signaling (from receptor/channel to calcium signaling to cell function), the advantages these alterations confer to cancer, and the best approaches for designing new calcium-focused therapies.

High expression levels of calcium channels, calcium pumps, and GPCRs (Table 1; protein or mRNA measurements) have been reported in patient breast cancer tissues over normal tissue (STIM1/2 (Miao et al. 2019), ORAI3 (Faouzi et al. 2011), SPCA2 pumps (Feng et al. 2010), P2X7 channels (Tan et al. 2015), TRPA1/TRPC1/TRPC3/TRPC6/TRPC7/TRPV6/TRPM7/TRPM8 channels (Aydar et al. 2009; Bolanz et al. 2008; Chodon et al. 2010; Dhennin-Duthille et al. 2011; Guilbert et al. 2008, 2009; Liu et al. 2014; Meng et al. 2013; Takahashi et al. 2018; Tsavaler et al. 2001), IP_3_Rs2/3 (Singh et al. 2017b), S100 proteins (Cross et al. 2005)) and high expression is correlated with breast tumor grade (RYRs (Abdul et al. 2008), TRPV6 channels (Dhennin-Duthille et al. 2011; Peters et al. 2012), TRPM8 channels (Yapa et al. 2018), TRPV4 channels (Peters et al. 2017), SPCA1 pumps (Grice et al. 2010), ORAI1 (McAndrew et al. 2011), P_2_Y6 GPCRs (Azimi et al. 2016), PMCA2 pumps (Peters et al. 2016; VanHouten et al. 2010), mitochondrial calcium uniporter (MCU) (Curry et al. 2013), S100 proteins (McKiernan et al. 2011)). Cancer patient samples over normal samples have also shown increased expression of CREB1/2 (Chhabra et al. 2007; Fan et al. 2012; Sofi et al. 2003), PKCζ (Paul et al. 2015; Smalley et al. 2019), and CAMKII (Chi et al. 2016), increased nuclear localization of NFAT2 (Quang et al. 2015), and increased phosphorylation of PKCζ (Paul et al. 2015), CAMKII (Chi et al. 2016), and CREB2 (Fan et al. 2012). High expression was also associated with poor survival for S100 (McKiernan et al. 2011), CAMKII (Chi et al. 2016), CREB1 (Chhabra et al. 2007), PKCα (Lonne et al. 2010), PMCA2 pumps (VanHouten et al. 2010), STIM1 (McAndrew et al. 2011), P_2_Y6 (Azimi et al. 2016), and TRPV6/TRPM7 channels (Middelbeek et al. 2012; Peters et al. 2012). In contrast, other reports find low expression in breast cancer tissues over normal tissue for VGCCs (Phan et al. 2017) and SERCA3 (Papp and Brouland 2011) or low expression correlated with breast tumor grade for VGCCs (Pera et al. 2016). Other evidence shows that high expression in breast cancer over normal tissue (Wang et al. 2015) and poor survival for VGCC gene alterations (Jacquemet et al. 2016), highlighting conflicting data for VGCCs.

**Table 1:** Overexpression of calcium-related proteins in human breast cancer patient samples. A list of differentially expressed calcium-related proteins in breast cancer patient tissue at the protein or transcript level is shown. Comparisons are made with respect to malignant vs. non-malignant tissue, tumor grade, and survival outcomes

	Protein	Comparison	Method	Reference
Calcium Channels	STIM1/2 STIM1	Malignant > adjacent High expression predicts poor survival	IHC mRNA	Miao et al. 2019 McAndrew et al. 2011
	ORAI1 ORAI3	Correlated with basal subtype Malignant > Normal	mRNA mRNA	McAndrew et al. 2011 Faouzi et al. 2011
	RyR	Correlated with tumor grade	IHC	Abdul et al. 2008
	IP3R2/3	Malignant > normal	IHC/mRNA	Singh et al. 2017b
	MCU	Correlated with basal subtype	mRNA	Curry et al. 2013
	P2X7	Malignant > normal	Western blot	Tan et al. 2015
	TRPA1	Malignant > normal	IHC/mRNA	Takahashi et al. 2018
	TRPC1/C6 TRPC3/C6 TRPC6 TRPC7	Malignant > normal Malignant > normal Malignant > normal Malignant > adjacent	IHC/mRNA mRNA IHC/mRNA mRNA	Dhennin-Duthille et al. 2011 Aydar et al. 2009 Guilbert et al. 2008 Tsavaler et al. 2001
	TRPM7	Malignant > normal Malignant > normal High expression predicts poor survival Malignant > normal	IHC/mRNA mRNA mRNA IHC/mRNA	Guilbert et al. 2009 Meng et al. 2013 Middelbeek et al. 2012 Dhennin-Duthille et al. 2011
	TRPM8	Malignant > normal Malignant > adjacent Correlated with basal subtype Malignant > normal	IHC Western blot/mRNA mRNA IHC/mRNA	Chodon et al. 2010 Liu et al. 2014 Yapa et al. 2018 Dhennin-Duthille et al. 2011
	TRPV4	Correlated with basal subtype	mRNA	Peters et al. 2017
	TRPV6	Malignant > normal Correlated with basal subtype High expression predicts poor survival Malignant > normal	mRNA mRNA mRNA IHC/mRNA	Bolanz et al. 2008 Dhennin-Duthille et al. 2011/Peters et al. 2012 Peters et al. 2012 Dhennin-Duthille et al. 2011
GPCRs	P2Y6	Correlated with basal subtype High expression predicts poor survival	mRNA mRNA	Azimi et al. 2016 Azimi et al. 2016
Calcium Pumps Calcium Effectors and Enzymes	PMCA2	Correlated with basal subtype High expression predicts poor survival	mRNA mRNA	Peters et al. 2016/VanHouten et al. 2010 VanHouten et al. 2010
	SPCA2	Malignant > adjacent Correlated with basal subtype	mRNA mRNA	Feng et al. 2010 Grice et al. 2010
	CREB CREB1 CREB1 CREB2	Malignant > adjacent Malignant > normal High expression predicts poor survival Malignant > normal	mRNA IHC/mRNA mRNA IHC/Western blot	Sofi et al. 2003 Chhabra et al. 2007 Chhabra et al. 2007 Fan et al. 2012
	CAMKII	Malignant > adjacent High expression predicts poor survival	IHC mRNA	Chi et al. 2016 Chi et al. 2016
	NFAT2	Malignant > normal	IHC	Quang et al. 2015
	PKCα	High expression predicts poor survival	mRNA	Lonne et al. 2010
	PKCζ	Malignant > normal Malignant > normal	IHC IHC/Western Blot	Paul et al. 2015 Smalley et al. 2019
	S100A6/A9/A11 S100A8/A9/A10/A11/A14 S100A11/A14	Malignant > normal Correlated with tumor grade High expression predicts poor survival	IHC mRNA mRNA	Cross et al. 2005 McKiernan et al. 2011 McKiernan et al. 2011

Similarly, human breast cancer cell lines were tested for expression differences. High expression (protein or mRNA measurements) has been reported in breast cancer cell lines compared with normal breast cell lines for SPCA2 pumps (Feng et al. 2010), PMCA1/2 pumps (Lee et al. 2002, 2005), ORAI1/3 channels (Faouzi et al. 2011; McAndrew et al. 2011), TRPC3/TRPC6/TRPM8/TRPV6 channels (Aydar et al. 2009; Liu et al. 2014; Peters et al. 2012), P_2_X4/5/7 channels (Jelassi et al. 2011, 2013), and P_2_Y2/P_2_Y6 GPCRs (Jin et al. 2014; Zhang et al. 2017), as well as increased phosphorylation of CAMKII (Chi et al. 2016) and CREB2 (Fan et al. 2012). Furthermore, NFATc2 (Kim et al. 2018), CREB (Son et al. 2010), and calreticulin (Lwin et al. 2010) showed higher expression in highly metastatic and tumorigenic cells over mildly tumorigenic cells. Expression differences are less clear for IP_3_Rs due to conflicting reports (Mound et al. 2017; Singh et al. 2017a), for RYRs and STIM due to lack of data, and for VGCCs due to variable expression patterns (Jacquemet et al. 2016).

In general, the patient and cell line data suggest that breast cancer elevates intracellular calcium concentration and signaling based on the overexpression of various plasma membrane calcium channels (P_2_X, TRP), intracellular release mechanisms (P_2_Y, RYR, IP_3_R), intracellular calcium store re-fill proteins (STIM/ORAI), and decreased expression of intracellular calcium store pumps (SPCA, SERCA). This notion of overactive calcium signaling is further supported by over expression and activation of calcium effectors (NFAT, CREB, CAMK, PKC). Of note, overexpression of PMCA may either conflict with these ideas (since it pumps cytoplasmic calcium outside of the cell) or it may serve to protect cancer cells from calcium overload. However, such broad conclusions would need to be supported by actual measurements of intracellular calcium concentrations or signaling. More detailed information such as spatial and frequency characteristics of the calcium signal may also be needed. In addition, whether these associations between increased expression and breast cancer are mere correlations or whether they have functional impact on breast cancer is necessary to test.

Indeed, some studies have investigated the functional impact of expression differences by targeting expression experimentally in breast cancer cells (Table 2). Increased expression of ORAI3 proteins found in cancer cells over normal cells was observed, and experimentally reducing the expression of ORAI3 inhibited cell proliferation and cell viability in cancer cells but not normal cells (Faouzi et al. 2011). Moreover, knockdown of ORAI1 or STIM1 in breast cancer cells led to reductions in in vitro migration and invasion and in vivo metastasis (Yang et al. 2009). Highly metastatic and tumorigenic cells show greater expression of IP_3_R3s over mildly tumorigenic cells, and knockdown of IP_3_R3s inhibited migration to a much greater extent in the aggressively tumorigenic and metastatic cells (Mound et al. 2017). Similarly, IP_3_Rs expression differences between cancer cells and normal cells was observed, but effects of downregulation of IP_3_R2/3s was only tested in cancer cells which reduced cancer cell viability (Singh et al. 2017a). Observations that cancer cells show increased phospho-CAMKII over normal cells guided investigators to test further overexpression of WT or phosphomimic CAMKII in cancer cells which enhanced colony formation, migration, and invasion (Chi et al. 2016). The increased expression of SPCA2 measured in cancer cells over normal cells was targeted for knockdown resulting in reduced cancer cell proliferation and colony formation (Feng et al. 2010). Likewise, high expression of TRPM8 channels in cancer cells over normal cells was targeted for knockdown to show reductions in cancer cell migration (Liu et al. 2014).

**Table 2:** Effect of calcium-related protein knockdown. A list of calcium-related proteins used in studies to test in parallel the effects of knockdown on both cellular function and calcium signaling is shown. The rationale is based on the overexpression of calcium channels, GPCR, calcium pumps, and calcium effectors and enzymes seen in patient tissue and aims to link cellular functional outcomes with changes in calcium signaling

	Protein knockdown	Functional outcome	Associated calcium signaling alteration	Reference
Calcium Channels	ORAI3 ORAI3 ORAI1	Inhibited in vitro proliferation and viability in MCF-7 Inhibited in vivo tumor growth in MCF-7 Reduced in vitro proliferation and viability in MCF-7 and MDA-MB-231	Reduced SOCE – Reduced SOCE	Faouzi et al. 2011 Motiani et al. 2013 McAndrew et al. 2011
	ORAI1 or STIM1	Reduced in vitro migration and invasion, and in vivo metastasis in MDA-MB-231	Reduced SOCE	Yang et al. 2009
	STIM1	Inhibited in vivo tumor growth and metastasis in MDA-MB-231	–	Miao et al. 2019
	IP3R3	Inhibited in vitro migration in MDA-MB-231	Switched from ATP-stimulated global intracellular calcium signal to an oscillating one	Mound et al. 2017
	IP3R2/3	Reduced in vitro viability in MCF-7	–	Singh et al. 2017a
	TRPM7	Reduced in vitro migration and in vivo metastasis in MDA-MB-231 Reduced in vitro viability in MCF-7	– Reduced resting calcium concentrations	Middelbeek et al. 2012 Guilbert et al. 2009
	TRPM8	Reduced in vitro migration in MDA-MB-231	–	Liu et al. 2014
	TRPV6	Reduced in vitro viability in T47D Reduced in vitro migration and invasion in MDA-MB-231 Reduced in vitro viability in T47D	– – Reduced TRPV6 calcium flux	Bolanz et al. 2008 Dhennin-Duthille et al. 2011 Peters et al. 2012
	MCU	Reduced in vivo tumor growth and metastasis in MDA-MB-231 Reduced in vitro migration in MDA-MB-231	Reduced ATP-stimulated mitochondrial calcium uptake Reduced SOCE	Tosatto et al. 2016 Tang et al. 2015
	VGCC	Reduced in vitro invasion in MDA-MB-231 Reduced in vitro proliferation in MCF-7	– –	Jacquemet et al. 2016 Taylor et al. 2008
Calcium pumps	PMCA2	Reduced in vitro proliferation in MDA-MB-231	–	Peters et al. 2016
	SPCA2	Reduced in vitro proliferation and colony formation in MCF-7	Reduced resting calcium concentrations	Feng et al. 2010
GPCRs	P2Y2	Reduced in vivo primary tumor growth and metastasis in MDA-MB-231 Reduced in vivo primary tumor growth and metastasis, and in vitro migration and invasion in MDA-MB-231	– Reduced ATP-stimulated cytosolic calcium signaling	Zhang et al. 2017 Jin et al. 2014
Calcium effectors and enzymes	PKCζ	Reduced in vitro migration and invasion and in vivo metastasis in MDA-MB-231	–	Paul et al. 2015, Smalley et al. 2019
	NFAT	Reduced in vitro invasion in MDA-MB-231 Reduced in vitro migration and invasion, and in vivo tumor growth in 4T1	– –	Kim et al. 2018 Quang et al. 2015
–	–	MCF-7 vs MCF10A	Increased resting calcium concentrations	Jonathan Pottle et al. 2013
–	–	HBL100 vs SKBR3 MCF10A vs MDA-MB-231	Reduced capacity to release calcium from intracellular stores but a more robust calcium entry during SOCE	Baldi et al. 2003 Motiani et al. 2010

Experimental reductions in many other different calcium signaling related proteins can inhibit the tumorigenic and invasive capacity for breast cancer cells (Table 2). The knockdown of P_2_Y2 GPCRs was able to reduce in vivo primary tumor growth and metastatic lesions (Jin et al. 2014; Zhang et al. 2017) and in vitro invasion and migration (Jin et al. 2014), STIM1 could inhibit tumor growth and metastasis (Miao et al. 2019), PKCζ reduced in vitro migration and invasion and in vivo metastasis (Paul et al. 2015; Smalley et al. 2019), TRPM7 channels led to decreased in vitro migration and in vivo metastasis (Middelbeek et al. 2012), NFAT was able to reduce in vitro invasion (Kim et al. 2018) and in vivo tumor growth (Quang et al. 2015), TRPV6 (Bolanz et al. 2008) or TRPM7 (Guilbert et al. 2009) reduced cell viability, VGCCs reduced cell invasion (Jacquemet et al. 2016), TRPV6 reduced migration/invasion (Dhennin-Duthille et al. 2011), ORAI3 inhibited in vivo tumor growth (Motiani et al. 2013), MCU decreased in vivo tumor growth and metastasis (Tosatto et al. 2016), and proliferation of breast cancer cells was reduced when VGCCs (Taylor et al. 2008) or PMCA2 (Peters et al. 2016) were targeted for knockdown. These data show that many calcium channels, pumps, GPCRs, and effectors are necessary for the migratory, invasive, proliferative, tumorigenic, or metastatic capacity of cancer cells. These data also bolster the expression data previously outlined for breast cancer patients and cells, which suggests that high expression of calcium channels, pumps, GPCRs, and effectors is utilized by breast cancer for survival advantages.

Finally, some experiments set out to determine differences in breast cancer calcium signaling by directly measuring intracellular calcium (Table 2). Basal intracellular calcium concentrations are higher in breast cancer cells over normal cells (Jonathan Pottle et al. 2013). Cancer cells over normal cells also exhibit a reduced capacity to release calcium from intracellular stores but a more robust calcium entry during SOCE (Baldi et al. 2003; Motiani et al. 2010). Experimental reductions in expression of ORAI1/3 not only reduced cell proliferation and viability but also inhibited SOCE in cancer cells (Faouzi et al. 2011; McAndrew et al. 2011). Similarly, SOCE could be reduced with knockdown of ORAI1 or STIM1 in breast cancer cells, but which also led to reductions in in vitro migration and invasion and in vivo metastasis (Yang et al. 2009). Breast cancer cell viability could be reduced by targeting TRPM7, which was linked to reductions in resting intracellular calcium concentrations (Guilbert et al. 2009). SPCA2 knockdown resulted in reduced resting calcium concentrations as well as reduced cancer cell proliferation and colony formation (Feng et al. 2010). Knockdown of MCU channels led to decreases in cancer cell migration and SOCE (Tang et al. 2015). Reductions in cell viability and calcium influx via TRPV6 occurred after TRPV6 knockdown (Peters et al. 2012). IP_3_R3s knockdown led to decreased cancer cell migration that was also associated with a switch from an ATP-stimulated global intracellular calcium signal to an oscillating one (Mound et al. 2017). P_2_Y2 GPCR knockdown was able to reduce ATP-stimulated cytosolic calcium signaling, as well as in vivo primary tumor growth and metastatic lesions, and in vitro invasion and migration (Jin et al. 2014). Reductions in cancer cell MCU expression was able to reduce ATP-stimulated mitochondrial calcium uptake and decreased in vivo tumor growth and metastasis (Tosatto et al. 2016). Overexpression of PMCA2 was able to protect cancer cells from ionomycin-stimulated calcium overload and apoptosis (VanHouten et al. 2010). In contrast to the many examples cited above, reductions in cancer cell proliferation via PMCA2 knockdown could not be attributed to any changes in cytosolic calcium signaling (Peters et al. 2016). In general, these data suggest that breast cancer cells not only rely on these calcium-related proteins for migratory, invasive, proliferative, tumorigenic, or metastatic behaviors but also rely on the associated intracellular calcium signals. However, future studies may reveal even more complexity beyond mere changes in protein expression, as genomically unstable cancer cells inherently have a high probability for mutagenesis that could alter protein activity and calcium signaling. Indeed, some studies report that cancer cell lines which harbor many different mutations, as well as specific cancer mutations such as KRas, can alter breast epithelial calcium signaling responses (Pratt et al. 2018, 2020).

## Conclusion

**Fig. 3:**
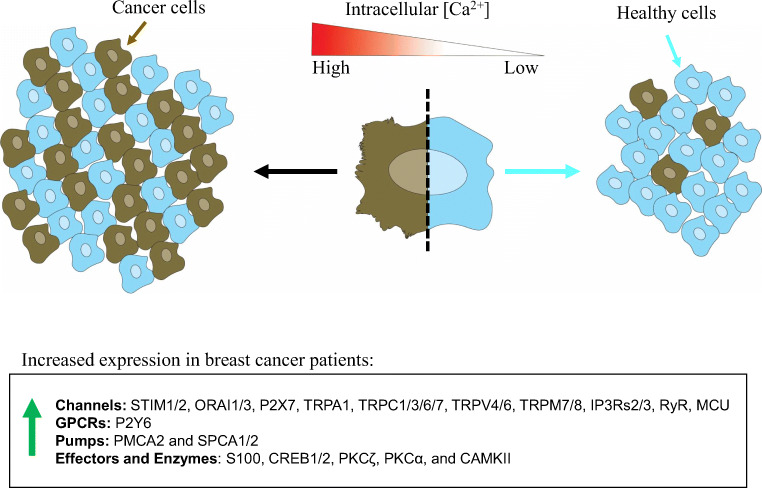
Calcium signaling is altered in breast cancer. The general hypothesis of abnormally elevated calcium signaling in the pathogenesis of breast cancer is illustrated. In general, human patient and cell line data suggest that breast cancer tumors and cells have high concentrations of intracellular calcium and/or an elevated capacity to mobilize calcium. This is based on the overexpression of various plasma membrane calcium channels (P2X, TRP), intracellular release mechanisms (P2Y, RYR, IP3R), intracellular calcium store re-fill proteins (STIM/ORAI), and calcium store pumps (PMCA, SPCA). This is further supported by overexpression and activation of calcium effectors (NFAT, CREB, CAMK, PKC). Moreover, experimental data using overexpression or knockdown of many calcium channels, pumps, GPCRs, and effectors in cells show that they are necessary for the migratory, invasive, proliferative, tumorigenic, or metastatic capacity of breast cancer cells. Finally, some data measuring intracellular calcium signaling directly suggest that breast cancer cells rely not only on the calcium-related proteins but also the associated intracellular calcium signals

The collective data reviewed here highlights the potential significance for cancer-dependent calcium signaling alterations in the progression of cancer. In conjunction, the patient and cell line data support an idea that breast cancer overexpresses calcium channels, pumps, GPCRs, and effectors, which are altering intracellular calcium signaling and ultimately aiding in migratory, invasive, proliferative, tumorigenic, or metastatic behaviors. The data suggest that breast cancer tumors and cells have high concentrations of intracellular calcium and/or an elevated capacity to mobilize calcium (Fig. 3). Still, more questions remain. While numerous components of the calcium signaling pathway are disrupted in cancer cells, the specific targets that will improve cancer treatment have not yet been fully clarified. It is also worth remembering that preclinical studies in mice do not always translate to success in the treatment of human disease. Clinical trials using therapies targeting calcium signaling or calcium-related proteins will be the clearest tests. Furthermore, there may be an opportunity to repurpose existing drugs, as combination therapy of simvastatin and doxorubicin show promising results in preclinical work, which additionally establishes a calcium-based therapeutic mechanism (Abdoul-Azize et al. 2018). Still, it is unclear which therapeutic approach in targeting calcium signaling for treating human breast cancer patients will be effective, since there are many potential targets as outlined above. Finally, the measured changes in calcium signaling with genetic ablation of calcium-related proteins are still somewhat correlative. More direct approaches are needed for modulating intracellular calcium and establish whether it can affect cancer cell behavior or tumor growth, in order to fully comprehend how breast cancer is altering intracellular calcium signaling as a selective advantage for survival and progression.
